# Cognitive bias modification Utilised to Rectify Errors for Depression (CURED): a double-blind, parallel-group feasibility randomised controlled trial in adults with depression

**DOI:** 10.1186/s13063-026-09668-6

**Published:** 2026-04-06

**Authors:** Kaan Alp Karamanlı, Jenny Yiend, Salma Al-Jaboby, Iulia Ceachir, Carolina Fialho, Zeqi Jia, Jessica John, Sarah Markham, Hannah Moloney, Tanya Ricci, Emre Ünal, Qixuan Wei, Sze Yin Wong, Sukhi Shergill, Jonas Everaert

**Affiliations:** 1 Department of Psychosis Studies, King’s College London, Institute of Psychiatry, Psychology & Neuroscience (IoPPN), 16 De Crespigny Park, London, SE5 8AF UK; 2 Institute of Psychiatry, Psychology & Neuroscience (IoPPN), King’s College London, London, UK; 3 Department of Biostatistics & Health Informatics, Institute of Psychiatry, Psychology & Neuroscience, King’s College, London, UK; 4 National Institute for Health and Care Research, Maudsley Biomedical Research Centre, London, UK; 5 Department of Psychiatry, Republic of Türkiye Ministry of Health, Reyhanlı State Hospital, Hatay, Türkiye; 6 Kent and Medway Medical School (KMMS), Canterbury, UK; 7 Department of Medical and Clinical Psychology, Tilburg University, Tilburg, The Netherlands; 8 Research Group of Quantitative Psychology and Individual Differences, KU Leuven, , Leuven, Belgium

**Keywords:** Depression, Anxiety, Cognitive bias modification for interpretation, CBM-I, Interpretation bias modification, CBM-errors, Randomised controlled trial, Feasibility trial, Digital mental health intervention, mHealth

## Abstract

**Background:**

Depression is a leading cause of global disability, yet access to evidence-based treatments such as CBT remains limited due to shortages of trained clinicians. Scalable, low-cost interventions offer a potential solution. Cognitive Bias Modification for Interpretation (CBM-I) is a promising candidate that is grounded in cognitive theory and backed by evidence. However, effects in depression have been weaker. To address previous limitations, we recently developed CURED (Cognitive bias modification Utilised to Rectify Errors for Depression), a six-session intervention co-developed with individuals with lived experience of depression and refined with clinician input. This study aims to evaluate the feasibility, acceptability and safety of CURED.

**Methods:**

This study is a double-blind, parallel-group feasibility randomised controlled trial recruiting 60 adults meeting inclusion criteria for clinical depression. Participants are allocated 1:1 either to the CURED intervention or a neutral CBM-I control matched for format, length, and delivery, though differing in content. Both arms involve six weekly sessions delivered via mailed pen-and-paper booklets, with remote researcher support to ensure fidelity, and assessment at baseline, post-treatment (6 weeks) and 12-week follow-up. Primary outcomes are feasibility (response, recruitment, eligibility, randomisation, retention, weekly assessment completion, adherence, and randomisation rates); acceptability (Credibility and Expectancy Questionnaire, Exit Questionnaire, and qualitative interviews); integrity of blinding; and safety (adverse and serious adverse events). Secondary outcomes include interpretation bias, depressive and anxiety symptoms, cognitive content and processes, and psychosocial functioning. Analyses will summarise feasibility metrics descriptively and provide variance estimates to inform sample size calculations and primary outcome selection for a future definitive trial.

**Discussion:**

CURED has the potential to address the pressing need for accessible, evidence-based interventions for depression. This feasibility trial will establish whether CURED is feasible, acceptable, and safe. If successful, a fully powered, definitive efficacy randomised controlled trial will be warranted.

**Trial registration:**

ISRCTN51689816, https://www.isrctn.com/ISRCTN51689816. Prospectively registered on 16 June 2025. Recruitment started on 16 July 2025 and is anticipated to be completed by 1 July 2026.

## Introduction

### Background and rationale {9a}

Depression stands out as one of the leading causes of global disability regardless of decades of intervention efforts [1], with a steadily increasing prevalence over the past three decades [2]. Although evidence-based approaches such as cognitive-behavioural therapy (CBT) are endorsed as gold-standard treatments by the National Institute for Health and Care Excellence (NICE) and the National Institute of Mental Health (NIMH), many individuals remain unable to access these treatments [3]. The main barrier appears to lie in the shortage of adequately trained clinicians [3]. This highlights the pressing need for scalable, accessible interventions.

**Fig. 1:**
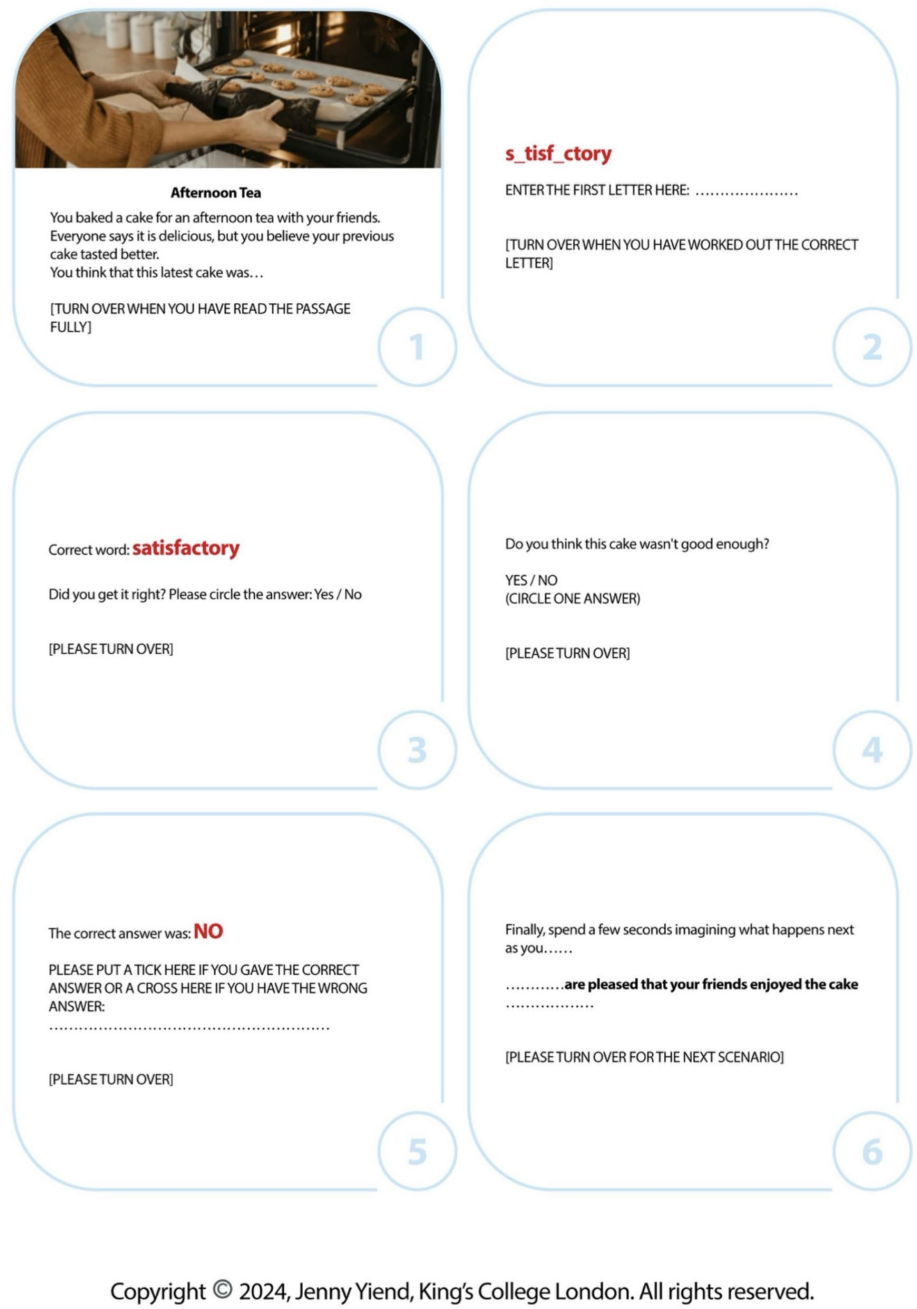
An example CURED item

Cognitive bias modification for interpretation (CBM-I), rooted in cognitive theory [4, 5] and supported by an expanding body of evidence [6, 7], emerges as a promising candidate. CBM-I is designed to shift the interpretation of emotionally ambiguous stimuli away from negative meaning and toward benign or positive interpretations [8]; see Fig. 1 for an example CBM-I item. The central role of interpretation bias (i.e. the mechanism targeted by CBM-I) has been consistently demonstrated in depression by meta-analyses on cross-sectional [9], longitudinal [10], and experimental studies modifying interpretation bias to reduce depressive symptoms [6]. Despite these findings, CBM-I appears to perform worse for depression compared to anxiety [6].

CBM-I was originally designed for anxiety; typical therapeutic content reflects core fears and processes specific to anxiety rather than depression [11]. Cognitive bias modification for cognitive errors (CBM-errors) was developed to rectify cognitive distortions in line with the cognitive thinking patterns (so called “errors”) originally identified and defined in Beck’s cognitive theory of depression [4, 12]. As well as targeting the full spectrum of depressive cognitions, training items are derived directly from clinical practice to provide maximum depression relevance [11, 12]. Early studies yielded encouraging preliminary findings [11], yet gains did not reliably extend to symptom improvement, underscoring the need to overcome existing limitations to achieve full therapeutic impact.

Insufficient number of sessions (i.e. one to two sessions) and disorder-specificity of the content stand out as the main limitations of CBM-Errors [12]. Indeed, the wider evidence base suggests that higher effect sizes can be obtained when a larger number of sessions [13] and disorder-specific therapeutic content is used [14, 15].

To address these limitations, we extended CBM-errors into a six-session intervention called CURED (cognitive bias modification utilised to rectify errors for depression) by generating new content co-created with people with lived experience of depression. New materials were evaluated by lived-experience experts and clinicians specialising in depression (Karamanli KA, Al-Jabboby S, Bayly-Bureau L, Fialho C, Everaert J, Hsu CW, et al: Beyond CBM-errors: the material development and evaluation of CURED (Cognitive bias modification Utilised to Rectify Error for Depression), In preparation). Evidence regarding feasibility, acceptability, and variance estimates is now needed to further CURED’s clinical translation pathway. This paper reports the protocol for a double-blind, parallel-group feasibility randomised controlled trial that aims to evaluate the feasibility, acceptability and safety of CURED.

### Explanation for the choice of comparator {9b}

We will employ a well-established control condition, validated in multiple prior CBM-I studies [16, 17]. This condition matches the intervention in all procedural features, delivery format, number and length of scenarios, structural format, and imagery instructions, with the sole difference being that the content focuses on emotionally neutral, everyday activities and factual descriptions. By holding other factors constant such as reading time, sentence length, delivery modality, guided imagery, and the word-completion task, this design isolates rectifying cognitive errors as the critical experimental variable.

### Objectives {10}

The specific objectives are to provide the following:

1. Feasibility metrics
2. Acceptability metrics
3. Safety assessment
4. Assessment of candidate outcome measures
5. Providing descriptive data and variance estimates for future trials

## Methods: patient and public involvement, and trial design

### Patient and public involvement {11}

Intervention materials were co-developed with patient and clinician involvement. Ten members of Maudsley Biomedical Research Centre’s Service Users Advisory Group (SUAG) generated 200 ambiguous daily-life scenarios across seven domains (e.g. work, study, relationships, leisure, health, and social interactions), following written guidance on cognitive bias modification and key cognitive error types including overgeneralisation, personalisation, catastrophising, dichotomous (“all-or-nothing”) thinking, and discounting the positive. The research team refined these into 181 standardised items, each comprising three lines of ambiguous text, a positive resolution (final word with missing vowels), a title, and a comprehension question. These newly developed items were added to previous CBM-Error content to create a total set of 282 intervention items. Control items (*n* = 240) were adapted from previous work on the STOP intervention, as the same neutral scenarios can serve as control materials across CBM-I paradigms [17]. All items were evaluated for readability by eight individuals with lived experience of depression and for negativity by eight clinicians with 6 months of therapeutic experience in depression. Finally, lived-experience co-author SM, the FAST-R panel, and King’s YPMHAG reviewed the protocol and participant-facing documents.

### Trial design {12}

Participants (Table 1) will be randomised to CURED or the placebo control procedures with assessments carried out at baseline (pre-randomisation); 6 weeks (immediately post-intervention); and 12-week follow-up. Primary outcomes will be collected at baseline, post-intervention, and 12-week follow-up, while a subset of measures will be administered after each weekly session throughout the 6 weeks of the intervention. A detailed overview of study procedures is provided in the CONSORT flow diagram (Fig. 3) and SPIRIT participant timetable (Table 2).

**Table 1:** Characteristics for the population planned to be sampled

**Characteristic**	**The people we would expect to see included**
Age	Anyone aged 18 or above
Sex	Sex at birth information is collected for randomisation purposes (see {*21b*} for details)
Gender	All are included. Male, female, non-binary, prefer not to say, and other (please specify) options are available for participants to select
Race, ethnicity and ancestry	The study uses the UK Census standard framework for data collection regarding race, ethnicity, and ancestry. There are no inclusion or exclusion criteria related to race, ethnicity, and ancestry
Socioeconomic status	Socioeconomic status is assessed using two items from the demographic questionnaire: 1. Highest level of education completed: No formal education/primary education/secondary education/associate degree/bachelor’s degree/master’s degree/doctorate/other (specify) 2. Annual household income: Less than £20,000/£20,000–£39,999/£40,000–£59,999/£60,000–£79,999/£80,000–£99,999/£100,000 or more There are no inclusion or exclusion criteria related to socioeconomic status
Geographic location	IoPPN, King’s College London (London, UK), however no in-person attendance is needed to participate. Any participant resident in England or Wales can participate. Participants must have access to an internet connection to complete the study procedures remotely
Other characteristics relevant to the trial	Mild self-reported depression or above determined by scoring above threshold on the Beck Depression Inventory-II (≥ 15) and answering “yes” to either or both of two MINI Screen depression questions

**Table 2:** The SPIRIT participant timeline of the CURED feasibility trial

	**Screening (-T**_**1**_** to T**_**0**_**)**	**Baseline (T**_**0**_**)**	**Interim assessments (T**_**i1-5)**_^**c**^	**6-week follow-up assessment **(**T**_**1**_**)**	**12-week follow-up (T**_**2**_**)**
Enrolment					
* Informed consent procedures*					
Participant information sheet	X				
Consent form	X				
*Eligibility check*					
Inclusion/exclusion criteria checklist	X				
Capacity assessment tool (if applicable)	X				
M.I.N.I. screening tool	X				
Beck depression inventory–II (BDI-II)	X	X		X	X
Intervention/comparator					
CURED (intervention)					
Neutral CBM-I (control)					
Assessment instruments					
*Sociodemographic information*					
Socio-demographic questionnaire		X			
*Clinical symptoms*					
M.I.N.I. MDD module		X			
Patient Health Questionnaire-9 (PHQ-9)		X	X	X	X
Beck Depression Inventory-II (BDI-II)		X		X	X
Depression, Anxiety, and Stress Scale-21 (DASS-21)		X		X	X
Generalized Anxiety Disorder-7 (GAD-7)				X	X
*Interpretation bias*					
Scrambled Sentence Task (SST)		X C/bal^b^	X C/bal	X C/bal	X C/bal
Similarity Rating Test (SRT)		X C/bal		X C/bal	X C/bal
*Cognitive content*					
Automatic Thought Questionnaire-Revised (ATQ-R)		X		X	X
Cognitive Distortions Scale (CDS)		X		X	X
*Prospective Imagery Task (PIT)*		X		X	X
*Cognitive process*					
Repetitive Thinking Questionnaire (RTQ-10)		X		X	X
Interpretation Inflexibility Task (IIT)		X		X	
***Psychosocial functioning and mood***					
Recovery of Quality of Life-20 (ReQoL-20)		X		X	X
Visual Analogue Scale (VAS) for mood (happy, sad, anxious)			X		
Global rating of change (GRC): 1 item				X	
***Feasibility measures***					
Credibility/Expectancy Questionnaire (CEQ)		X		X	
Exit Questionnaire					X

^a^Arrow indicates continuous delivery of the assigned condition

^b^C/bal stands for counterbalancing, which was used alternate item content across multiple timepoints to prevent order effects. The specific counterbalancing schedule is available upon request

^c^The interim (i.e. weekly) assessments are completed only after completing the intervention/control session of that week

## Methods: participants, interventions, and outcomes

### Trial setting {13}

The study is conducted by the Institute of Psychiatry, Psychology & Neuroscience (IoPPN), King’s College London, co-sponsored by South London and Maudsley NHS Trust. Assessments are delivered as online questionnaires (Qualtrics links) via email, and intervention or control booklets are posted weekly to participants, who return them in one batch at the end of the 6-week period in a pre-paid envelope. Participants are asked to complete a screening call, baseline interview, and subsequently their assigned intervention and associated assessments in a private setting, without distractions where possible.

### Characteristics of the people who are needed for the trial

#### Eligibility criteria for participants {14a}

The inclusion criteria are the following:

1. Having mild depression or above, assessed by:
  - Scoring ≥ 15 (mild) on Beck Depression Inventory-II (BDI-II) [18] AND
  - Answering “yes” to either or both of two depression questions (Section A, questions 1 and 2) in the Mini-International Neuropsychiatric Interview Screen (M.I.N.I.; [19]).
2. Age 18 years or over.

The exclusion criteria are the following:

1. Any medical condition that impedes the completion of the study tasks (e.g. advanced cancer, serious heart disease, stroke, dementia);
2. Having received psychological therapy targeting the same cognitive mechanisms as the intervention in the 2 months leading up to participation;
3. If on psychotropic medication, having changed medication or dose within the month prior to participating in the study or expected to do so during the study;
4. Psychosis, according to screening item of the Modified MINI Screen;
5. Substance or alcohol misuse operationalised as ≥ 2 total score on CAGE questions Adapted to Include Drug Use (CAGE-AID; [20]);
6. Self-reported Autism Spectrum Disorder diagnosis
7. A previous head injury resulting in a loss of consciousness;
8. Reporting suicidal thoughts with current intent, as assessed by BDI-II item 9 and follow-up Columbia-Suicide Severity Rating Scale (C-SSRS; [21]**)** questions 3 and 4, with exclusion if answering “yes” to question 4;
9. Participating in any other interventional study concurrently.

### Eligibility criteria for sites and those delivering interventions {14b}

This trial will be conducted at a single site under the lead of the Chief Investigator (JY), a site investigator (KK; a clinical researcher and PhD candidate), and the Project Coordinator (CF). Recruitment and intervention delivery will be undertaken by a team comprising post-graduate research assistants (RAs), MSc students, and the clinical researcher (KK).

### Who will take informed consent? {32a}

Capacity to consent will be presumed in line with the Mental Capacity Act (2005) unless in doubt, in which case assessed by researchers during screening using a tool based on the Act to determine whether the individual can understand, retain, and communicate information about the study and their participation. Informed consent will be obtained by trained members of the recruitment team (postgraduate MSc students, graduate research assistants, and the site investigator), using secure eConsent forms sent with the participant information sheet at least 24 h in advance, and copies are stored securely on the university’s password-protected servers.

### Additional consent provisions for collection and use of participant data and biological specimens {32b}

We will include an explicit item in our informed consent procedures explaining the process for fully anonymised data sharing via the national repository, UK Data Archive. Only participants who select this option will be included in the archived dataset.

## Intervention and comparator

### Intervention and comparator description {15a}

#### Intervention condition (CURED)

The CURED intervention comprises six sessions, each containing 40 three-line scenarios presented in a booklet format. In each scenario, the ambiguity is resolved by a positive word fragment at the end of the passage, followed by a comprehension question (for a full description of the training procedure, see [12], pp. 150–151). Finally, participants generate prospective imagery related to the passage for 8 s [22]. An example intervention item is shown in Fig. 1.

#### Control condition (neutral CBM-I)

**Fig. 2:**
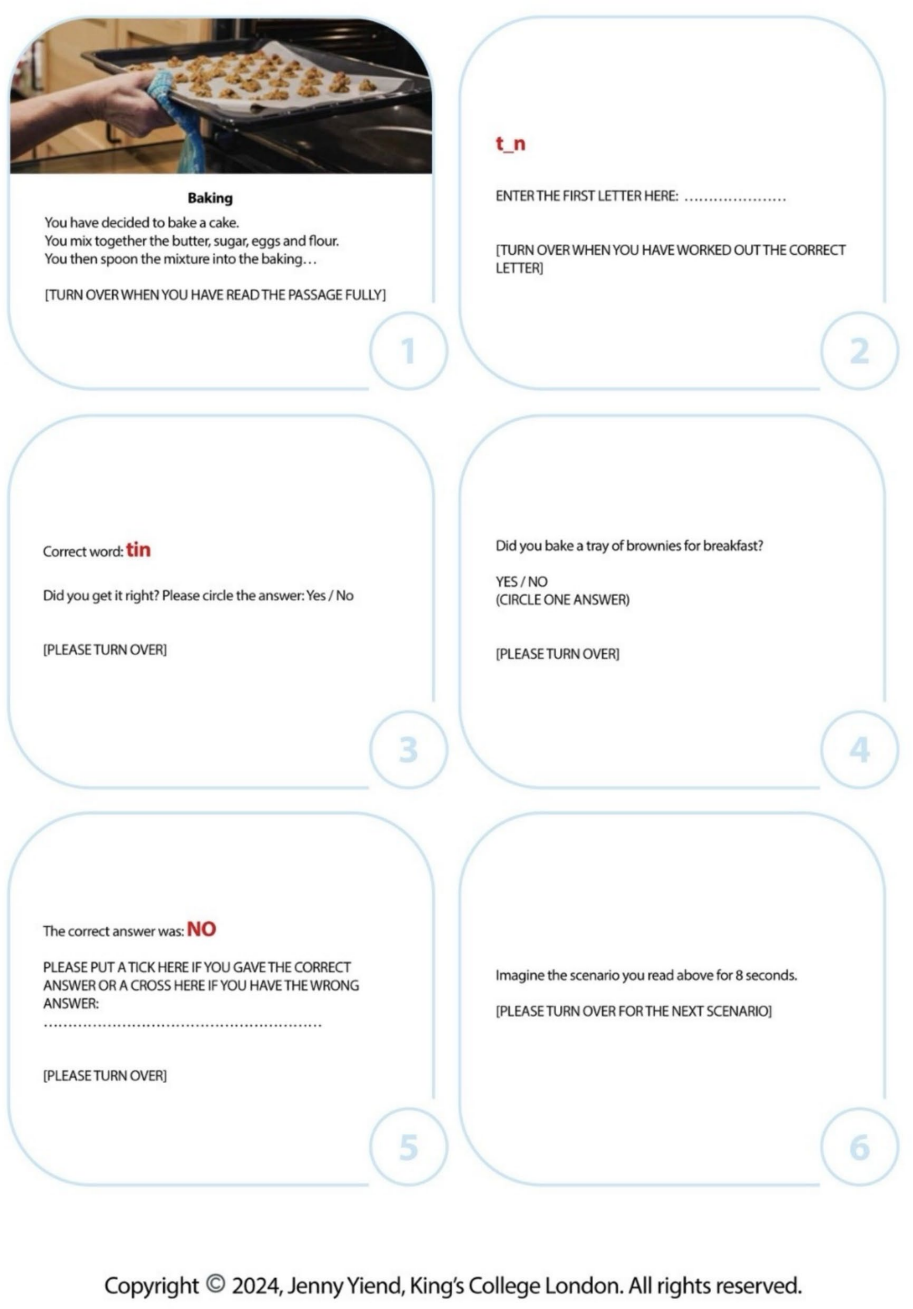
An example control condition item

The control condition also comprises six sessions of booklets with 40 three-line scenarios in each session, employing the exact same procedural flow as the intervention. It matches the intervention in delivery, number and length of scenarios, structural format, and imagery instructions, with the sole difference being that the content focuses on emotionally neutral, everyday activities and factual descriptions (please see Fig. 2 item as an example).

### Criteria for discontinuing or modifying allocated intervention/comparator {15b}

Participants may discontinue or be discontinued if adverse events (AEs), serious adverse events (SAEs), or mood worsening are identified at any point during the study and deemed related to the study procedures. This is assessed using an adverse events checklist (see Harms {17} for more information).

### Strategies to improve adherence to intervention/comparator {15c}

Adherence to the allocated intervention or control condition will be supported and monitored through structured participant contact. At the first session, a researcher will conduct an orientation call to explain procedures and address any questions. Weekly email reminders will be sent to encourage ongoing engagement, with additional reminders if sessions are missed. A scheduled check-in call will take place at week 3 (mid-point) to assess mood, review progress, troubleshoot difficulties, and reinforce adherence. Researchers will also conduct calls at each main assessment point (baseline, post-intervention, and 12-week follow-up) to support participation and ensure data completeness.

### Concomitant care permitted or prohibited during the trial {15d}

Participants receiving psychotherapy involving cognitive components are eligible only after a 2-month washout period following the end of therapy. Where antidepressant or other psychotropic medication has been recently initiated or adjusted, eligibility is deferred for 1 month after medication stabilisation. During recruitment and follow-up calls, researchers emphasise that participant wellbeing is the priority and that involvement in this trial should not delay or prevent access to clinical care. Participants who initiate psychotherapy or medication change after randomisation will not be excluded from the trial.

### Ancillary and post-trial care {34}

No specific ancillary or post-trial care is necessary as part of this feasibility trial; participants will continue with their usual treatment.

### Outcomes {16}

#### Primary outcomes

As this study is a feasibility evaluation of the CURED intervention, there is no single primary clinical outcome. Instead, (1) feasibility, (2) acceptability, (3) blinding integrity, and (4) safety will be assessed across several domains. These metrics will inform progression criteria for a future definitive RCT.

1. *Feasibility* will be quantified using response, recruitment, eligibility, and randomisation rates; adherence; retention; and weekly assessment completion rate.
2. *Acceptability* will be assessed using quantitative measures (Credibility and Expectancy Questionnaire; Exit Questionnaire) and qualitative interviews exploring user experience, barriers, and facilitators to engagement.
3. *Blinding integrity* will be evaluated by the success of randomisation, percentage of unblindings, and by examining whether participants and researchers can correctly guess allocation at the final 12-week follow-up (last contact point).
4. *Safety* will be evaluated throughout the trial by monitoring and documenting adverse events (AEs) and serious adverse events (SAEs), with frequency, severity, and relatedness recorded at each assessment point.

#### Secondary outcomes

The secondary outcomes will assess the suitability of candidate clinical and cognitive measures as potential primary outcomes in a future definitive RCT. This includes evaluating their sensitivity to change over time and estimating between-group effect sizes (with 95% confidence intervals) to inform sample size calculations (please see Table 2 for the full assessment schedule).

##### Target mechanism (interpretation bias) measures

**Scrambled sentences test (SST **[23, 24]**)**

A 12-item cognitive task involving rearrangement of six-word scrambled sentences into grammatically correct statements while under time pressure.

**Similarity Rating Test (SRT **[12]**)**

A validated measure of interpretation bias in which participants read ten ambiguous scenarios followed by sentences reflecting positive and negative interpretations, rating each for similarity to the original passage.

##### Clinical symptom measures

**Mini-International Neuropsychiatric Interview (M.I.N.I. **[19]**)—MDD module**

A structured diagnostic interview used to determine whether participants meet DSM/ICD criteria for major depressive disorder at baseline.

**Beck Depression Inventory-II (BDI-II **[18]**)**

A 21-item self-report questionnaire assessing the severity of depressive symptoms on a 0–3 scale, with higher scores indicating greater depression severity.

**Patient Health Questionnaire-9 (PHQ-9 **[25, 26]**)**

A 9-item self-report scale measuring depressive symptoms on a 0–3 scale, widely used for tracking symptom severity and change over time.

**Depression, Anxiety, and Stress Scale–21 (DASS–21 **[27]**)**

A 21-item self-report measure assessing depression, anxiety, and stress, scored on a 0–3 scale to yield three subscale totals.

**Generalised Anxiety Disorder-7 (GAD-7 **[28]**)**

A 7-item self-report questionnaire evaluating anxiety severity on a 0–3 scale, with higher scores reflecting greater anxiety.

#### Exploratory measures

A set of cognitive content and process measures will be included to examine potential mechanisms of change and generate hypotheses for future work.

##### Cognitive content measures

**Automatic Thoughts Questionnaire–Revised (ATQ-R **[29]**)**

A 40-item self-report measure of automatic thoughts relevant to depression, comprising 30 negative and 10 positive items rated on a 5-point scale to yield total frequency scores of negative thinking.

**Cognitive Distortions Scale (CDS **[30]**)**

A 20-item questionnaire assessing 10 types of cognitive distortions across interpersonal and achievement domains, rated on a 7-point scale indicating how often each distortion occurs.

**Prospective Imagery Task (PIT **[31]**)**

A 20-item task assessing the vividness of positive and negative future-oriented mental imagery, with higher ratings indicating more vivid prospective imagery.

##### Cognitive process measures

**Repetitive Thinking Questionnaire (RTQ-10 **[32]**)**

A 10-item self-report scale measuring the tendency to engage in repetitive, transdiagnostic negative thinking following distressing events.

**Interpretation Inflexibility Task (IIT **[33]**)**

A picture-based paradigm assessing interpretation bias and flexibility across three progressively clarified social scenarios, with higher scores reflecting greater interpretive flexibility.

##### Psychosocial functioning and mood monitoring

**Recovering Quality of Life (ReQoL-20 **[34]**)**

A 20-item self-report measure of recovery-oriented quality of life, where higher total scores indicate better well-being and functioning.

**Visual Analogue Scales (VAS) for mood (happy, sad, anxious)**

Three single-item scales assessing momentary levels of happiness, sadness, and anxiety on a 0–100 continuum, with higher scores indicating greater intensity.

**Global Rating of Change (GRC **[35]**)**

A single-item measure of perceived overall change in health status since baseline, indicating whether participants feel worse, the same, or better.

### Harms {17}

The intervention paradigm, cognitive bias modification for interpretation (CBM-I), has been evaluated in over 100 trials and has consistently been found to be safe and no riskier than standard care [7]. The primary potential harm is temporary distress during task completion for the intervention group when reading content which could trigger depressive thoughts. Prior feasibility data on the predecessor of CURED, CBM-Errors, indicated that participants’ immediate mood was typically unaffected or slightly improved following sessions [12]. Adverse Events (AEs) information will be proactively collected at the 3-week check-in call using an adapted version of the Adverse Events Checklist, and an exit interview will provide a further opportunity for adverse event data gathering. If a participant experiences persistent mood deterioration, distress impairing daily functioning, or any indication of risk to self or others, participation will be paused, a risk assessment made and, if appropriate, clinical care will be arranged (e.g. referral to the participant’s GP or mental health team). All AEs judged to be serious, according to the standard HRA definition, will be reported to the Sponsor and REC in line with governance requirements. All adverse events will be presented descriptively by frequency, relatedness to the intervention, severity and seriousness to provide comprehensive safety data which will be reported in trial outputs.

### Participant timeline {18}

**Fig. 3:**
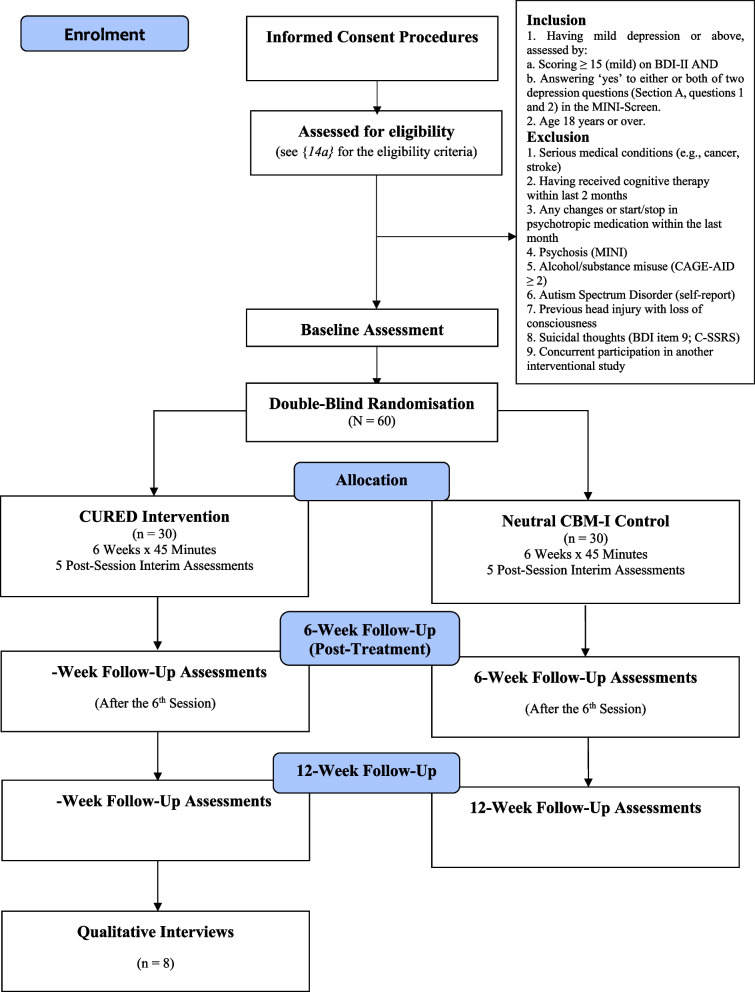
The CONSORT flow diagram of the feasibility trial procedure

Table 2 and Fig. 3 describe the participant flow in the trial.

### Sample size {19}

As this is a feasibility trial, formal power analysis was not appropriate. Consistent with recommendations that feasibility studies include approximately 25–35 participants per arm [36–38], we set our target sample size at 30 participants per arm (60 participants in total).

### Recruitment {20}

Potential participants will be identified through a) South London and Maudsley (SLaM) Consent for Contact (C4C) register, after permission is gained for direct approach from the relevant clinician b) advertising (e.g. King’s College London curricular research newsletter; SLaM’s *Take Part in Research* newsletter) c) the McPin Foundation and d) self-referral via the study website (www.curedtrial.co.uk). For routes b-d potential participants will be asked to email a dedicated study enquiry address (curedresearchenqueries@kcl.ac.uk). Upon receiving an enquiry, the recruitment team will respond by sending the Participant Information Sheet (PIS) and inviting the individual to schedule an initial research call for consent and screening, ensuring at least 24 h for consideration. Those meeting eligibility criteria during this call will then be scheduled for an online baseline assessment, after which participants will be randomised. Participants will receive the relevant intervention materials by post at the start of the week following randomisation.

## Assignment of interventions: randomisation

### Sequence generation: who will generate the sequence {21a}

The random allocation sequence will be generated and overseen by a randomisation team (CF, JY, EU) who are not part of the patient-facing research team and not otherwise involved in trial delivery. The randomisation team is responsible for creating and maintaining the randomisation file and communicating identifier codes.

### Sequence generation: type of randomisation {21b}

Stratified block randomisation will be used to allocate participants to intervention or control arms using an excel spreadsheet. Stratification will be based on sex at birth (male/female) and number of previous depressive episodes (0/1/2 +).

### Allocation concealment mechanism {22}

The allocation sequence will be stored in a password-protected randomisation Excel file accessible only to unblinded randomisers. The intervention materials are prepared by randomisers in sealed opaque envelopes labelled only with the Participant Identification Number (PIN) and week number. Sessions are returned en masse at the end of a participant’s assigned intervention using pre-paid envelopes addressed to the randomisers, who are the only team members authorised to open and securely store them. This process ensures full concealment of allocation until after trial completion.

### Implementation {23}

Blinded researchers contact the randomisers by email notifying them of a new enrolment. Randomisers assign the next available PIN and provide the relevant sealed envelopes to researchers for weekly posting. Sealed envelopes are then posted by the blinded researchers to participants according to the weekly schedule.

## Assignment of interventions: blinding

### Who will be blinded {24a}

The randomisation team (CF, JY, EU) will be unblinded. All other team members are blinded.

### How will be blinding be achieved {24b}

Blinded team members will see only sealed envelopes and will be unable to access the randomisation files which are password protected and stored in a separate online domain from the remainder of the trial administration files, whose access is restricted to the randomisation team. Blinded team members will see only sealed envelopes and will be unable to access the randomisation files, which are password protected and stored in a separate online domain from the remainder of the trial administration files, whose access is restricted to the randomisation team.

Participants are requested not to disclose their assigned condition to the researchers. Participants will open and complete their booklets remotely and without interaction with the researchers. Should a researcher become unblinded, for example by a participant disclosing their assigned condition during an assessment or check-up call, this will be recorded by the researcher in a protocol violation document.

### Procedure for unblinding if needed {24c}

Unblinding may be requested by the Chief Investigator or Sponsor. In the unlikely case of a team member becoming aware of a clinical emergency (e.g. serious self-harm, suicide, risk of harm to others, psychiatric hospital admission involving a participant) that could be related to the intervention or study procedures, or where there is uncertainty, the following steps will be taken:

1. Team member reports to the Chief Investigator within 24 h
2. Chief Investigator consults with the Sponsor, if needed, within 48 h
3. If unblinding is required, the participant’s allocation will be disclosed by the randomiser team accessing the randomisation file which holds details of the assigned condition
4. All instances of unblinding will be documented and reported to the Sponsor who advises on the need to report to the HRA.

## Data collection and management

### Plans for assessment and collection of outcomes {25a}

All clinical, cognitive, and feasibility outcomes will be assessed using validated instruments with well-established psychometric properties, as detailed in the Outcomes {10} section. With the exception of eligibility screening and the MINI MDD module (administered by a trained assessor via telephone), assessments are self-report questionnaires administered using Qualtrics, with participants completing the surveys online while remaining on a video or telephone call with a researcher.

Recruitment team members administering assessor-led measures (e.g. screening calls, MINI MDD) are trained through role-play and supervised to ensure fidelity and accuracy.

### Plans to promote participant retention and complete follow-up {25b}

Participants will receive reminder emails or text messages for each session and before each interim and main assessment, alongside sustained contact with the research team through regular calls at baseline, week 3, post-treatment (week 6), and the 12-week follow-up. To acknowledge participants’ time investment, reimbursement will be provided in line with their level of involvement: a total of £80, divided into four instalments of £20 following completion of (1) the baseline assessment, (2) the first three weekly assessments, (3) the post-treatment assessment, and (4) the 12-week follow-up assessment. Retention will also be supported by the research team remaining available during online assessments to address any technical or procedural questions without interfering in responses.

Participants will be retained in the study wherever possible, even if they initiate therapy or medication during the trial. Participants may opt to continue with outcome assessments only, as an alternative to complete study withdrawal.

### Data management {26}

This study will generate both quantitative (self-report questionnaires, experimental tasks at baseline, post-treatment, and 12-week follow-up) and qualitative data (semi-structured exit interviews at post-treatment) in both paper and electronic format. Electronic data will be stored on secure King’s College London servers, hosted through King’s cloud services. The main datasets will be prepared in both R and SPSS formats, with approximately 60 cases expected. Paper materials (i.e. booklets) will be stored in locked filing cabinets within secure university premises, accessible only to authorised members of the research team.

#### Data entry and quality assurance

For manually entered data (i.e. paper booklet data), a double-entry procedure will be implemented in SPSS. Two independent files will be cross-checked with an automated comparison program, and any inconsistencies will be investigated and corrected. Where relevant, data entry is automated via Qualtrics survey software. Quality will be monitored throughout by generating descriptive statistics and range checks (e.g. detecting implausible values such as out-of-range questionnaire scores or impossible demographic values such as age = 150).

#### Data standards

Data handling will follow the best-practice standards set out by the UK Data Archive, including systematic documentation of data and metadata. The study will be conducted in accordance with the FAIR principles [39]. Pre-registration of study design and analyses will ensure transparency.

#### Pseudonymisation and security

Each participant will be assigned a unique Participant Identification Number (PIN), used both on electronic records and paper booklets. All paper records will carry only participant identification numbers (PINs) and no personal identifiers. All datasets will be stored using these PINs, while the master list linking PINs to personal identifiers (name, contact details) will be kept separately on a secure drive, password-protected at the individual file level, and accessible only to the Chief Investigator (custodian of the trial data). During the active trial, data will remain pseudonymised; after analysis and UK data archive deposit, the master list will be destroyed (estimated August 2026), rendering the data fully anonymised.

#### Archiving and retention

Paper data will be digitised as soon as possible, and electronic copies will be stored on encrypted King’s College London servers. Source paper data will be archived in accordance with King’s Data Retention Schedules Policy.

In line with King’s Records and Data Retention Schedule, data will be retained for 5 years after project completion. After anonymisation, datasets will be deposited in the UK Data Archive (via the ReShare process) with accompanying metadata: a codebook, variable labels, provenance documentation, and a read-me file. An embargo of 12 months will be applied to allow publication of main trial outputs.

#### Governance and access

From 12 months after study completion the anonymised dataset will be openly accessible via UK Data Archive for those participants who have consented to data sharing.

### Confidentiality {33}

All information provided by participants will be treated in strict confidence, in accordance with the Data Protection Act (2018) and UK GDPR. The limits of confidentiality, including circumstances where disclosures may necessitate action (e.g. where a participant is considered at risk of serious harm to self or others), are explained in the participant information sheet and discussed during the informed consent process. During the trial, all participants will be assigned a unique Participant Identification Number (PIN) that will be used in place of personal identifiers on study materials and datasets. Personal identifiers (e.g. names, contact details) will be stored separately from research data on password-protected, access-restricted King’s College London servers. Only authorised members of the research team will have access to these identifiers, solely for the purposes of study coordination. After data collection, pseudonymised datasets will be stored securely and, following the destruction of the master list linking identifiers to PINs, will be fully anonymised before archiving and sharing. At no stage will individual participants be identifiable in study reports, publications, or presentations.

For qualitative data, transcripts of interviews will be pseudonymised during transcription by assigning PINs to the participants and removing or replacing any identifying information (e.g. names of people, places, or organisations). When direct quotes are used in publications or presentations, they will be carefully selected to avoid inclusion of any content that could potentially identify the participant.

## Statistical methods

### Statistical methods for primary and secondary outcomes {27a}

As this is a feasibility trial, formal hypothesis testing will not be undertaken, and no single primary clinical endpoint has been defined. Instead, analyses will focus on descriptive statistics and estimation.

#### Primary outcomes

1. Feasibility metrics
  - Response rate: N, percent, who progressed from initial referral to first direct contact.
  - Recruitment rate: N, percent, consenting.
  - Eligibility rate: N, percent, eligible (meeting inclusion and exclusion criteria after screening).
  - Randomisation rate: n, percent, randomised overall and per month against the target of 60 total, five per month.
  - Retention: N, percent, providing data at post-treatment and 12-week follow-up.
  - Assessment completion: N, percent, completion overall and per week throughout the intervention period.
  - Adherence: mean (SD) number of sessions completed out of six, and percentage completing ≥ 80 % (≥ 5/6).
2. Acceptability metrics
  - Credibility and Expectancy Questionnaire: mean (SD) scores summarised by group at baseline and post-treatment.
  - Exit Questionnaire: mean (SD) Likert-scale ratings on overall experience, manageability, and likelihood of future use at week 12.
  - Qualitative Interviews: with a purposive subsample (*n* = 8, intervention arm) varying in gender, depression severity, age, and ethnicity, analysed thematically to explore acceptability, barriers, facilitators, and delivery preferences.
3. Integrity of blinding
  - Success of randomisation and concealment summarised descriptively.
  - Percentage of unblindings reported as protocol violations
  - Analysis of participants’ guesses of allocation at 12-week follow-up.
4. Safety
  - Frequencies, severities, and relatedness of all AEs/SAEs by arm will be presented descriptively and tabulated.
  - Qualitative content analysis of AE reports will identify common categories and potential risk factors.

### Secondary outcomes

Secondary outcomes will be assessed as candidate primary outcomes and used in providing effect size estimates for power calculations for future trials.

1. Suitability of the candidate primary outcomes will be defined by:
  - Acceptability (feedback on burden and clarity from exit questionnaires),
  - Variability (sufficient spread in scores without floor/ceiling effects),
  - Sensitivity to change, defined as evidence of within-group and between-group score changes over time in the expected direction (mean change scores with 95% CIs).
2. Descriptive statistics will be aggregated by assigned condition and presented in tables, including means, medians, standard deviations, variances, range, 95% confidence intervals and levels of missingness.

#### Who will be included in each analysis {27b}

All available data will be included in the final aggregations, with sensitivity analysis excluding certain characteristics (e.g. low adherence, withdrawal, starting therapy or medication) where applicable.

#### How missing data will be handled in the analysis {27c}

Missingness will be reported for each variable, following CONSORT guidelines, including reasons for loss to follow-up, where known. We do not anticipate differential attrition between arms, as participants will be unaware of their allocation, though this will still be assessed by examining completion rates of assessments and assigned sessions across arms separately. To examine potential bias, baseline characteristics will be compared between participants with complete versus missing follow-up data within each arm. Missingness will also be summarised both as proportions per variable and graphically (ranking variables from most to least complete). Predictors of missingness will be examined across the entire sample and by arm to inform missing at random assumptions in a full trial.

#### Methods for additional analyses (e.g. subgroup analyses) {27d}

As this is a feasibility trial, no formal subgroup analyses are planned. Descriptive tables may be prepared to examine whether feasibility and acceptability metrics (e.g. adherence, retention, exit questionnaire feedback) vary by baseline characteristics such as gender or depression severity.

#### Interim analyses {28b}

No interim analyses are planned.

#### Protocol and statistical analysis plan {5}

A statistical analysis plan (SAP) is integrated within this protocol (see Sections {27a–d}). No separate standalone SAP will be published, as is appropriate for a feasibility trial with primarily descriptive statistical methods. Should amendments be required, these will be documented and reported in academic publications in accordance with CONSORT and SPIRIT guidelines.

## Oversight and monitoring

### Composition of the coordinating centre and trial steering committee {3d}

The coordinating centre for this trial is the research team based at King’s College London, Department of Psychosis Studies, who are responsible for day-to-day management and operational support, including recruitment, randomisation, data collection, and data management.

A Project Management Group (PMG) will act in place of a formal Trial Steering Committee and provide both operational and higher-level oversight of the trial. The PMG is responsible for supervising trial conduct, reviewing study progress, and recommending amendments or actions where required. Weekly operational meetings are held to review recruitment progress, address questions or issues arising, and discuss topics requiring immediate attention. PMG meetings are attended by the randomisation team, recruitment team, and the Chief Investigator (CI), all based at King’s College London, and are jointly led by the CI, Project coordinator, and the clinical researcher site lead.

### Composition of the data monitoring committee, its role and reporting structure {28a}

N/A. A separate data monitoring committee (DMC) was not deemed necessary for this feasibility study because (1) the trial does not involve a medicinal product or a high-risk intervention, (2) it is an early-phase feasibility trial with a limited sample size, and (3) risks are minimal and continuously monitored by the Trial Management Group, under the oversight of the Sponsor, and the Research Ethics Committee. Therefore, in line with guidance for low-risk feasibility trials, a DMC was not required.

### Frequency and plans for auditing trial conduct {29}

Regular audits are carried out within the host organisation and by the sponsor, and if selected for audit, the study will cooperate with all procedures and make all files available. While no such audits are currently scheduled, the Sponsor reserves the right to initiate an audit at any stage, either in response to concerns regarding trial conduct, data integrity, or participant safety, or on a random basis. The PMG conducts regular informal audits by inspection of files and procedures and by shadowing activities of team members.

### Protocol amendments {31}

Any substantial protocol modification will be submitted to the REC for approval and communicated to the Sponsor before implementation. Minor administrative changes (e.g. corrections of typographical errors or clarifications) will also be documented in the protocol amendment log to maintain a complete version history. All protocol versions will be formally logged (e.g. Protocol v1.1, date) in a version-controlled record. Public trial registries (ISRCTN and/or ClinicalTrials.gov) will be updated promptly to reflect any amendments, ensuring transparency.

## Dissemination policy {8}

Results will be published in high quality academic journals and presented at appropriate national and international conferences, for example the annual British Association of Behavioural and Cognitive Psychotherapies conference, or the triennial World Confederation of Behavioural and Cognitive Psychotherapies (https://wccbt.org).

The study and its findings will also be publicised on the study website (www.curedtrial.co.uk), through the professional dissemination services provided by The McPin Foundation (https://mcpin.org/), circular newsletters provided by King’s SUAG and through the production of dissemination materials such as infographics, leaflets, podcasts, and articles for newsletters and popular press. These will be proactively disseminated through user networks, relevant mental health charity websites and newsletters, and NHS trust communications.

## Discussion

There is a pressing need for scalable, easy-access interventions for depression, given persistent barriers to accessing existing treatments [3]. The CURED intervention is well positioned to address this gap, as few low-intensity treatments for depression are both accessible and rigorously validated. Building on prior work on CBM-I approaches for depression (e.g. CBM-Errors; 12–14), CURED employs training materials co-developed and evaluated with input from lived-experience experts and clinicians. However, evidence on its feasibility and efficacy is lacking.

Feasibility testing is particularly important before a definitive efficacy trial, as it mitigates the risk of costly revisions or sunk costs that may arise from launching a full-scale randomised controlled trial (RCT) prematurely. Without such preparatory work, key design elements such as outcome selection and sample size estimates risk being arbitrary, potentially compromising trial efficiency and validity. By addressing these issues early, feasibility testing reduces the likelihood of downstream challenges in the translational pathway [40, 41].

Accordingly, the present early-stage, randomised, double-blind feasibility trial of CURED aims to generate essential evidence on recruitment, retention, adherence, safety and acceptability, as a precursor to a definitive RCT. Despite its limitations (e.g. a smaller sample size compared to a definitive trial, the paper-and-pen session administration), the trial will clarify procedures and progression criteria for subsequent research.

## Trial status

Recruitment began on 16/07/2025, with the first eligible participant randomised on 18/08/2025. Recruitment is estimated to continue until 01/07/2026. Please see the registration here: ISRCTN51689816, https://www.isrctn.com/ISRCTN51689816, registered on 16/06/2025. Protocol version is 1.2 (25/12/2024), approved by London–Fulham Research Ethics Committee on 13/03/2025 (REC reference: 24/LO/0855).

## Data Availability

No datasets were generated or analysed during the current study.

## Abbreviations

AE: Adverse event
ATQ-R: Automatic Thoughts Questionnaire–Revised
BADE: Bias Against Disconfirmatory Evidence
BDI-II: Beck Depression Inventory-II
CBM-I: Cognitive bias modification for interpretation
CBT: Cognitive behavioural therapy
CDS: Cognitive Distortions Scale
CEQ: Credibility and Expectancy Questionnaire
CI: Chief Investigator
CONSORT: Consolidated Standards of Reporting Trials
CURED: Cognitive bias modification utilised to rectify errors for depression
C4C: Consent for contact
DASS-21: Depression, Anxiety and Stress Scale-21
DMHI: Digital mental health intervention
FAST-R: Feasibility and Acceptability Support Team for Researchers
GAD-7: Generalised Anxiety Disorder-7
GRC: Global rating of change
ICC: Intraclass correlation coefficient
IIT: Interpretation inflexibility task
IoPPN: Institute of Psychiatry, Psychology & Neuroscience
MAR: Missing at random
MCID: Minimal clinically important difference
MHRA: Medicines and Healthcare products Regulatory Agency (UK)
MINI: Mini-International Neuropsychiatric Interview
NHS: National Health Service
NICE: National Institute for Health and Care Excellence
NIMH: National Institute of Mental Health
PHQ-9: Patient Health Questionnaire-9
PIN: Participant identification number
PIT: Prospective imagery task
PPI: Patient and public involvement
ReQoL-20: Recovering Quality of Life-20 Items
RMSSD: Root mean square of successive differences
RCT: Randomised controlled trial
RTQ-10: Repetitive Thinking Questionnaire-10
SAE: Serious adverse event
SAR: Serious adverse reaction
SLaM: South London and Maudsley NHS Foundation Trust
SPIRIT: Standard Protocol Items: Recommendations for Interventional Trials
SRT: Similarity Rating Test
SST: Scrambled Sentences Test
SUSAR: Suspected unexpected serious adverse reaction
SUAG: Service User Advisory Group
TMG: Trial Management Group
UK GDPR: United Kingdom General Data Protection Regulation
VAS: Visual Analogue Scale
YMPHG: Young Mental Health Group

## References

[1] World Health Organization. Depression and other common mental disorders: global health estimates. Geneva, Switzerland: World Health Organization; 2017. WHO/MSD/MER/2017.2.

[2] Moreno-Agostino D, Wu YT, Daskalopoulou C, Hasan MT, Huisman M, Prina M. Global trends in the prevalence and incidence of depression: a systematic review and meta-analysis. J Affect Disord. 2021;281:235–43. 33338841 10.1016/j.jad.2020.12.035

[3] Layard R, Clark DM. Why more psychological therapy would cost nothing. Front Psychol. 2015 [cited 2025 June 17];6. Available from: http://journal.frontiersin.org/Article/10.3389/fpsyg.2015.01713/abstract. 10.3389/fpsyg.2015.01713 PMC4658447 26635648

[4] Beck AT. Cognitive therapy and the emotional disorders. New York: International Universities Press; 1976.

[5] Mathews A, MacLeod C. Cognitive vulnerability to emotional disorders. Annu Rev Clin Psychol. 2005;1:167–95. 10.1146/annurev.clinpsy.1.102803.143916 17716086

[6] Fodor LA, Georgescu R, Cuijpers P, Szamoskozi Ş, David D, Furukawa TA, et al. Efficacy of cognitive bias modification interventions in anxiety and depressive disorders: a systematic review and network meta-analysis. Lancet Psychiatry. 2020;7(6):506–14. 32445689 10.1016/S2215-0366(20)30130-9

[7] Martinelli A, Grüll J, Baum C. Attention and interpretation cognitive bias change: a systematic review and meta-analysis of bias modification paradigms. Behav Res Ther. 2022;157:104180. 36037642 10.1016/j.brat.2022.104180

[8] Mathews A, Mackintosh B. Induced emotional interpretation bias and anxiety. J Abnorm Psychol. 2000;109(4):602–15. 11195984

[9] Everaert J, Podina IR, Koster EHW. A comprehensive meta-analysis of interpretation biases in depression. Clin Psychol Rev. 2017;58:33–48. 28974339 10.1016/j.cpr.2017.09.005

[10] Vos LMW, Nieto I, Amanvermez Y, Smeets T, Everaert J. Do cognitive biases prospectively predict anxiety and depression? A multi-level meta-analysis of longitudinal studies. Clin Psychol Rev. 2025;1:116-102552. 10.1016/j.cpr.2025.102552 39923703

[11] Lester KJ, Mathews A, Davison PS, Burgess JL, Yiend J. Modifying cognitive errors promotes cognitive well being: a new approach to bias modification. J Behav Ther Exp Psychiatry. 2011;42(3):298–308. 21352718 10.1016/j.jbtep.2011.01.001

[12] Yiend J, Lee JS, Tekes S, Atkins L, Mathews A, Vrinten M, et al. Modifying interpretation in a clinically depressed sample using ‘cognitive bias modification-errors’: a double blind randomised controlled trial. Cogn Ther Res. 2014;38(2):146–59.

[13] Menne-Lothmann C, Viechtbauer W, Höhn P, Kasanova Z, Haller SP, Drukker M, et al. How to boost positive interpretations? A meta-analysis of the effectiveness of cognitive bias modification for interpretation. Voracek M, editor. PLoS ONE. 2014 June 26;9(6):e100925. 10.1371/journal.pone.0100925 PMC4072710 24968234

[14] Beadel JR, Ritchey FC, Teachman BA. Role of fear domain match and baseline bias in interpretation training for contamination fear. J Exp Psychopathol. 2016 [cited 2025 Sept 26]. Available from: https://journals.sagepub.com/doi/abs/10.5127/jep.045414.

[15] Daniel EK, Johnco C, Sicouri G. Content-specificity of a single-session cognitive bias modification of interpretations for social anxiety. J Affect Disord Rep. 2025;1:21-100937.

[16] Salemink E, Kindt M, Rienties H, van den Hout M. Internet-based cognitive bias modification of interpretations in patients with anxiety disorders: a randomised controlled trial. J Behav Ther Exp Psychiatry. 2014;45(1):186–95. 24177146 10.1016/j.jbtep.2013.10.005

[17] Yiend J, Lam CLM, Schmidt N, Crane B, Heslin M, Kabir T, et al. Cognitive bias modification for paranoia (CBM-pa): a randomised controlled feasibility study in patients with distressing paranoid beliefs. Psychol Med. 2023;53:10-4614. 10.1017/S0033291722001520 PMC10388312 35699135

[18] Beck AT, Steer RA, Brown G. Beck Depression Inventory–II. APA PsycTests. 1996 [cited 2025 Oct 12]; Available from: 10.1037/t00742-000

[19] Sheehan DV, Lecrubier Y, Sheehan KH, Amorim P, Janavs J, Weiller E, et al. The Mini-International Neuropsychiatric Interview (M.I.N.I.): the development and validation of a structured diagnostic psychiatric interview for DSM-IV and ICD-10. J Clin Psychiatry. 1998;59 Suppl 20:22–33;quiz 34–57. 9881538

[20] Brown RL, Rounds LA. Conjoint screening questionnaires for alcohol and other drug abuse: criterion validity in a primary care practice. Wis Med J. 1995;94(3):135–40. 7778330

[21] Posner K, Brown GK, Stanley B, Brent DA, Yershova KV, Oquendo MA, et al. The Columbia-Suicide Severity Rating Scale: initial validity and internal consistency findings from three multisite studies with adolescents and adults. Am J Psychiatry. 2011;168(12):1266–77. 22193671 10.1176/appi.ajp.2011.10111704 PMC3893686

[22] Lee JS, Mathews A, Shergill S, Yiu Chan DK, Majeed N, Yiend J. How can we enhance cognitive bias modification techniques? The effects of prospective cognition. J Behav Ther Exp Psychiatry. 2015;49:120–7. 25841654 10.1016/j.jbtep.2015.03.007

[23] Wenzlaff RM. The mental control of depression: psychological obstacles to emotional well-being. In: Wegner DM, Pennebaker JW, editors. Handbook of mental control. Englewood Cliffs (NJ): Prentice-Hall; 1993. p. 239–257.

[24] Würtz F, Zahler L, Blackwell SE, Margraf J, Bagheri M, Woud ML. Scrambled but valid? The scrambled sentences task as a measure of interpretation biases in psychopathology: a systematic review and meta-analysis. Clin Psychol Rev. 2022;93:102133. 35219928 10.1016/j.cpr.2022.102133

[25] Zuithoff NP, Vergouwe Y, King M, Nazareth I, van Wezep MJ, Moons KG, et al. The Patient Health Questionnaire-9 for detection of major depressive disorder in primary care: consequences of current thresholds in a crosssectional study. BMC Fam Pract. 2010;11:1-98. 21144018 10.1186/1471-2296-11-98 PMC3009622

[26] Kroenke K, Spitzer RL, Williams JBW. The PHQ-9. J Gen Intern Med. 2001 Sept 1;16(9):606–13. 10.1046/j.1525-1497.2001.016009606.x PMC1495268 11556941

[27] Lovibond PF, Lovibond SH. The structure of negative emotional states: comparison of the Depression Anxiety Stress Scales (DASS) with the Beck Depression and Anxiety Inventories. Behav Res Ther. 1995;33(3):335–43. 7726811 10.1016/0005-7967(94)00075-u

[28] Spitzer RL, Kroenke K, Williams JBW, Löwe B. A brief measure for assessing generalized anxiety disorder: the GAD-7. Arch Intern Med. 2006;166(10):1092–7. 16717171 10.1001/archinte.166.10.1092

[29] Hollon SD, Kendall PC. Cognitive self-statements in depression: development of an automatic thoughts questionnaire. Cognitive Therapy and Research. 1980;4:383–95.

[30] Covin R, Dozois DJA, Ogniewicz A, Seeds PM. Measuring cognitive errors: initial development of the Cognitive Distortions Scale (CDS). Int J Cogn Ther. 2011;4(3):297–322.

[31] Blackwell SE, Rius-Ottenheim N, Schulte-van Maaren YWM, Carlier IVE, Middelkoop VD, Zitman FG, et al. Optimism and mental imagery: a possible cognitive marker to promote well-being? Psychiatry Res. 2013;206(1):56–61. 23084598 10.1016/j.psychres.2012.09.047 PMC3605581

[32] McEvoy PM, Thibodeau MA, Asmundson GJG. Trait repetitive negative thinking: a brief transdiagnostic assessment. J Exp Psychopathol. 2014;3:1–17.

[33] Deng W, Everaert J, Creighton M, Bronstein MV, Cannon T, Joormann J. Developing a novel assessment of interpretation flexibility: reliability, validity and clinical implications. Personality and Individual Differences. 2022;190:111548.

[34] Keetharuth AD, Brazier J, Connell J, Bjorner JB, Carlton J, Buck ET, et al. Recovering Quality of Life (ReQoL): a new generic self-reported outcome measure for use with people experiencing mental health difficulties. Br J Psychiatry. 2018;212(1):42–9. 29433611 10.1192/bjp.2017.10 PMC6457165

[35] Kamper SJ, Maher CG, Mackay G. Global rating of change scales: a review of strengths and weaknesses and considerations for design. J Man Manip Ther. 2009;17(3):163–70. 20046623 10.1179/jmt.2009.17.3.163 PMC2762832

[36] Sim J, Lewis M. The size of a pilot study for a clinical trial should be calculated in relation to considerations of precision and efficiency. J Clin Epidemiol. 2012;65(3):301–8. 22169081 10.1016/j.jclinepi.2011.07.011

[37] Teare MD, Dimairo M, Shephard N, Hayman A, Whitehead A, Walters SJ. Sample size requirements to estimate key design parameters from external pilot randomised controlled trials: a simulation study. Trials. 2014;15:264. 24993581 10.1186/1745-6215-15-264 PMC4227298

[38] Whitehead AL, Julious SA, Cooper CL, Campbell MJ. Estimating the sample size for a pilot randomised trial to minimise the overall trial sample size for the external pilot and main trial for a continuous outcome variable. Stat Methods Med Res. 2016;25(3):1057–73. 26092476 10.1177/0962280215588241 PMC4876429

[39] Wilkinson MD, Dumontier M, Aalbersberg IjJ, Appleton G, Axton M, Baak A, et al. The FAIR Guiding Principles for scientific data management and stewardship. Sci Data. 2016;3(1):160018. 10.1038/sdata.2016.18 PMC4792175 26978244

[40] Lyon AR, Koerner K. User‐centered design for psychosocial intervention development and implementation. Clin Psychol Sci Pract. 2016;23(2):180–200. 10.1111/cpsp.12154 PMC5812700 29456295

[41] Skivington K, Matthews L, Simpson SA, Craig P, Baird J, Blazeby JM, et al. A new framework for developing and evaluating complex interventions: update of Medical Research Council guidance. BMJ. 2021;374:n2061. 10.1136/bmj.n2061 PMC8482308 34593508

